# Random nanostructured metallic films for environmental monitoring and optical sensing: experimental and computational studies

**DOI:** 10.1186/s11671-015-0855-x

**Published:** 2015-03-27

**Authors:** Ivan Karbovnyk, John Collins, Ivan Bolesta, Andriy Stelmashchuk, Antonina Kolkevych, Shereen Velupillai, Halyna Klym, Orest Fedyshyn, Svitlana Tymoshuk, Ihor Kolych

**Affiliations:** Ivan Franko National University of Lviv, 1 Universytetska str, Lviv, 79000 Ukraine; Department of Physics and Astronomy, Wheaton College, 26 East Main Street, Norton, MA 02766 USA; Lviv Polytechnic National University, 12 Bandera str, Lviv, 79013 Ukraine

**Keywords:** Metal-dielectric films, Optical properties, Computer simulations

## Abstract

Nanostructured silver films are studied using computational and experimental methods. Surface plasmon resonance-related phenomena are emphasized. Resonant optical absorption band changes due to the influence of noxious gases are investigated. Amplification of light at the film surface due to local electromagnetic field enhancement at the nanoscale is discussed based on finite difference time domain calculations.

## Background

Novel engineered nanostructures which exhibit superior optical and other physical properties with respect to conventional materials offer many opportunities for the assessment of toxic air contaminants [1,2]. Recent developments, for example, include SO_2_ gas detector utilizing SnO_2_ nanoparticles as sensing elements [3] and ZnO-based nanostructured nitrogen dioxide sensor [4]. While sensitive, one drawback is that these sensors only operate at temperatures higher than 200°C.

A promising class of materials that can be extremely sensitive for environmental changes at ambient conditions is noble metal nanoparticles [5] and noble metal nanostructures, in which light-metal interaction induces surface plasmon resonance (SPR) [6].

Understanding of specific optical phenomena and their relation to structural characteristics in noble metal-based nanostructures is crucial for the design of efficient thin-film-based environmental sensors. According to numerous reports, resonant absorption peaks associated with surface plasmon excitations are expected to be very sensitive to environmental contaminants such as noxious gases [7,8]. On the other hand, the effect of local field enhancement is observed in these materials, which is sensitive to the dielectric environment of the metallic nanoparticles, and can be used for the amplification of light at the nanoscale [9].

Present research is focused on the experimental and computational studies of nanostructured silver films, having in mind these two perspectives.

## Methods

Random nanostructured films were grown by thermal deposition of silver onto dielectric substrate (glass) in vacuum (10^−6^ Torr). The deposition rate was around 0.5 nm per min. Film morphology was controlled by adjusting the parameters of the deposition process. Mass thickness of the film was monitored using quartz crystal resonator, the resonant frequency of which depends on the thickness of deposited film. Such technique is widely used for accurate control of deposition thickness and typically ensures measurement of film thickness to a precision of 1 nm. Prepared samples were examined with a Solver P47 PRO atomic force microscope (AFM) from NT-MDT, Zelenograd, Russia [10]. Obtained films contain both a conducting phase (silver clusters) and an insulating phase (cavities between clusters). Mass thicknesses of Ag coverage vary from 1 to 10 nm; 5-, 7-, and 8-nm films were chosen for optical experiments.

For the study of the influence of noxious gases on the optical properties of nanostructured films, samples were placed in chamber filled with carbon dioxide (CO_2_) or nitrogen dioxide (NO_2_) for fixed periods of time. Carbon dioxide was produced according to the following reaction:

1 $$ {\mathrm{CaCO}}_3+2\mathrm{H}\mathrm{C}\mathrm{l}\to {\mathrm{CaCl}}_2+{\mathrm{CO}}_2+{\mathrm{H}}_2\mathrm{O} $$

In order to obtain nitrogen dioxide, the reaction of copper with concentrated nitric acid was exploited:

2 $$ \mathrm{C}\mathrm{u}+{\mathrm{H}\mathrm{NO}}_3\to \mathrm{C}\mathrm{u}{\left({\mathrm{NO}}_3\right)}_2+{\mathrm{NO}}_2+{\mathrm{H}}_2\mathrm{O} $$

Optical absorption measurements were performed with ULAB S108UV spectrophotometer from ULAB, Kyiv, Ukraine, in the visible range from 350 to 750 nm. This instrument uses halogen lamp as a light source, Si detector, and a single beam optical system with grating monochromator (1,200 lines per mm). Spectral resolution is 1 nm at 0.3% photometric accuracy in transmittance mode. All spectra were collected at room temperature.

A nanostructured silver film model for computer simulations was implemented by defining a 3D simulation space containing empty volume pixels (voxels) and a substrate surface. A Monte Carlo iterative algorithm was used in order to determine voxels filled by silver particles.

A parallel 3D finite difference time domain (FDTD) solver was implemented to study the electromagnetic properties of nanostructured noble metal films [11,12]. The application has been created using C++ language using IDE Qt Creator. The algorithm is designed for parallel computing; therefore, OpenMP library for gcc compiler was exploited. Maximum number of threads used in the algorithm is eight. Simulations were run on Intel(R) Core(TM) i7 2.90GHz CPU powered machine. The propagation of a plane wave in the 3D computation domain with the dimension of 200 × 200 × 50 voxels and 1,000 timesteps takes approximately 1 min (depending on density of the film).

The permittivity of silver was modeled using Drude equation with parameters, described in [13]. We used the perfectly matched layer (PML) boundary conditions in FDTD method to prevent the reflections from boundaries of computed space while simulating plane wave propagation.

## Results and discussion

**Figure 1:**
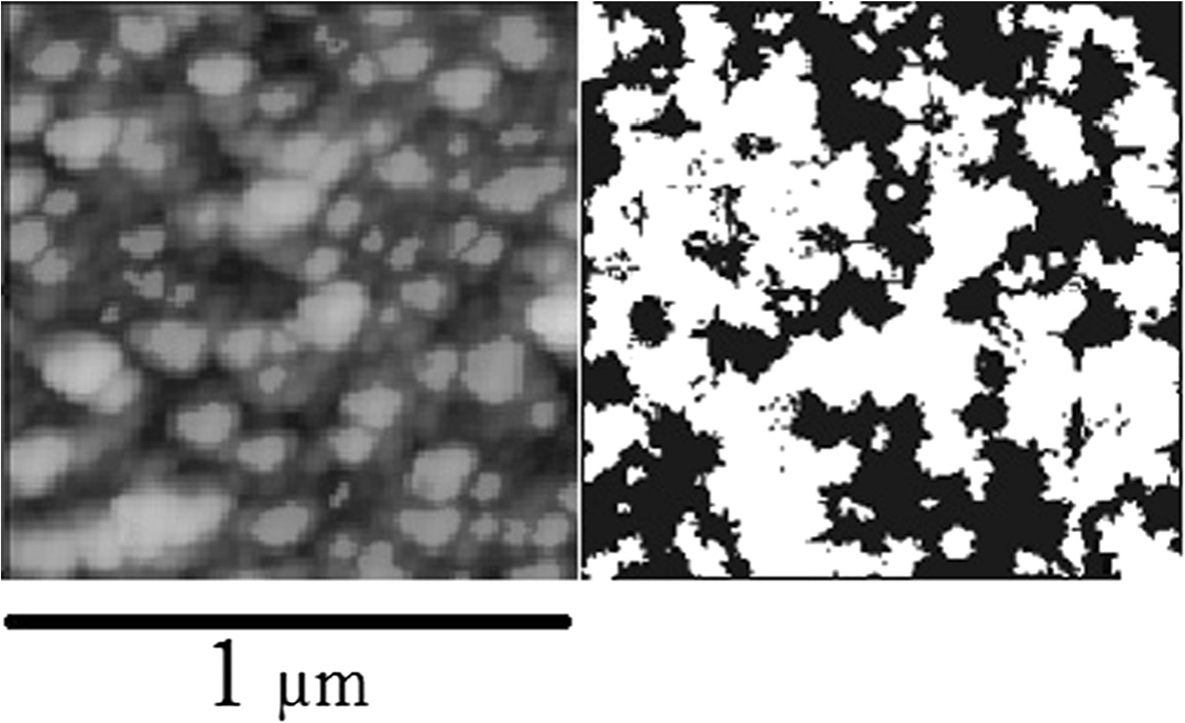
**Typical SEM micrograph of a near percolation silver film and the respective 2D topographic model.**

**Figure 2:**
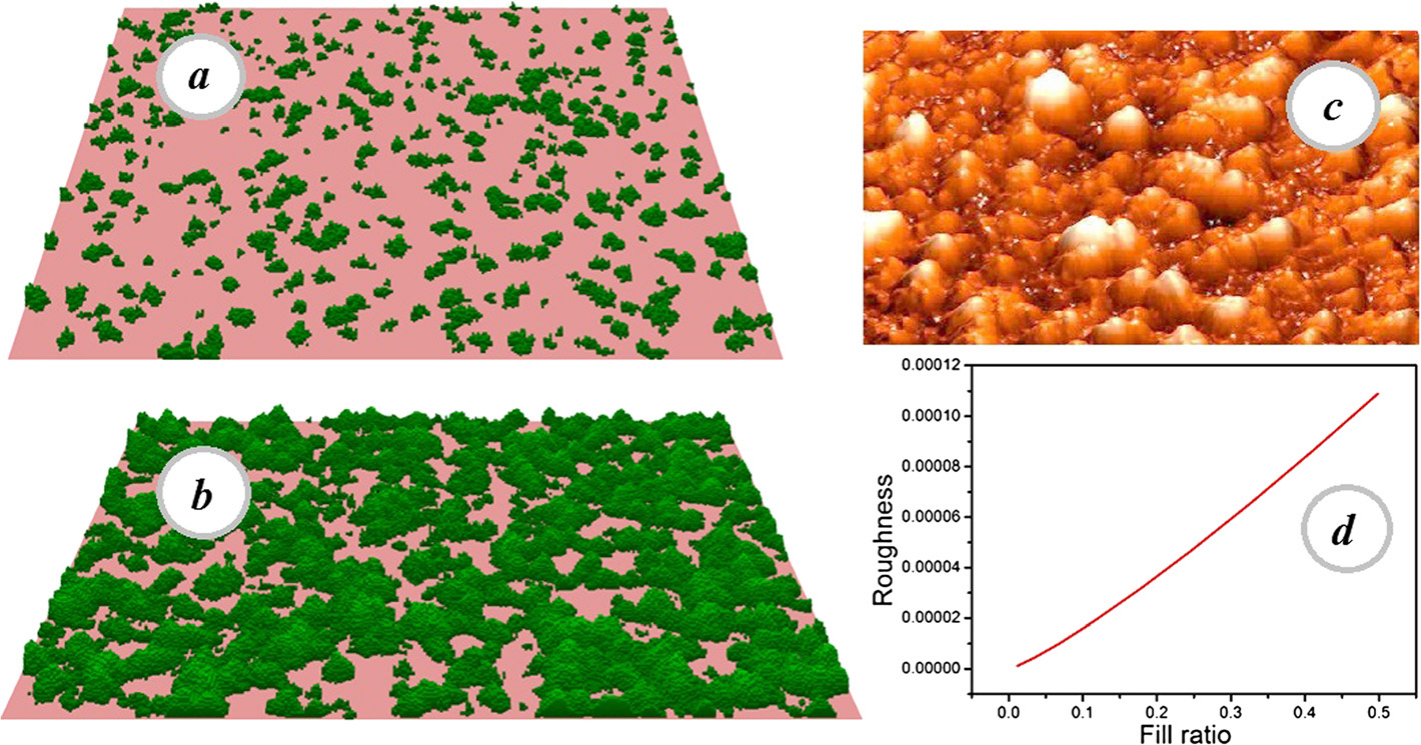
**3D computer-generated patterns and AFM image of a real structure.** Showing the morphology of island films (**(a)** 0.15 fill ratio, **(b)** 0.55 fill ratio); real morphology of the nanostructured silver film (height increases from darker to brighter, the brightest points correspond to approximately 15 nm) as seen by AFM **(c)**; simulated film roughness (mean square deviation of film height at each simulated point from the average film thickness) as a function of fill ratio **(d)**.

Figure 1 shows the fragment of a typical scanning electron microscopy (SEM) image of obtained near percolation silver film along with the two-dimensional computer-generated reconstruction. Properties of the films, including roughness and fractal dimension, were calculated for different simulated structures. Roughness was estimated at different iterations of the simulation algorithm. Fractal dimension of the simulated film was obtained by box counting [14] and falls within 2.0…2.5 range. Island structure of the simulated random films and the evolution of the film roughness with the increase of fill ratio are illustrated in Figure 2. For the purpose of comparison, real Ag film morphology observed using atomic force microscopy imaging is also shown.

In order to study possible local field intensity enhancements in nanostructured films, FDTD calculations were performed. The structural features of the simulated geometries were based on the above described computer-generated patterns. The size of the films used in these simulations was 200 × 200 nm. Two models with 0.15 and 0.55 fill factor were considered. Film thickness in simulations increases with fill factor. In our case, the linear dimension of an elementary block of the film is 1 nm. Hence, the average thickness of simulated metal islands is 2.5 nm for the 0.15 fill factor and 3.9 nm for the 0.55 fill factor. Maximum thickness of the film in the simulation can reach up to 5 nm and up to 10 nm for 0.15 and 0.55 fill factors, respectively.

The intensity map was calculated in a plane just inside the glass substrate and below the film/substrate interface.

**Figure 3:**
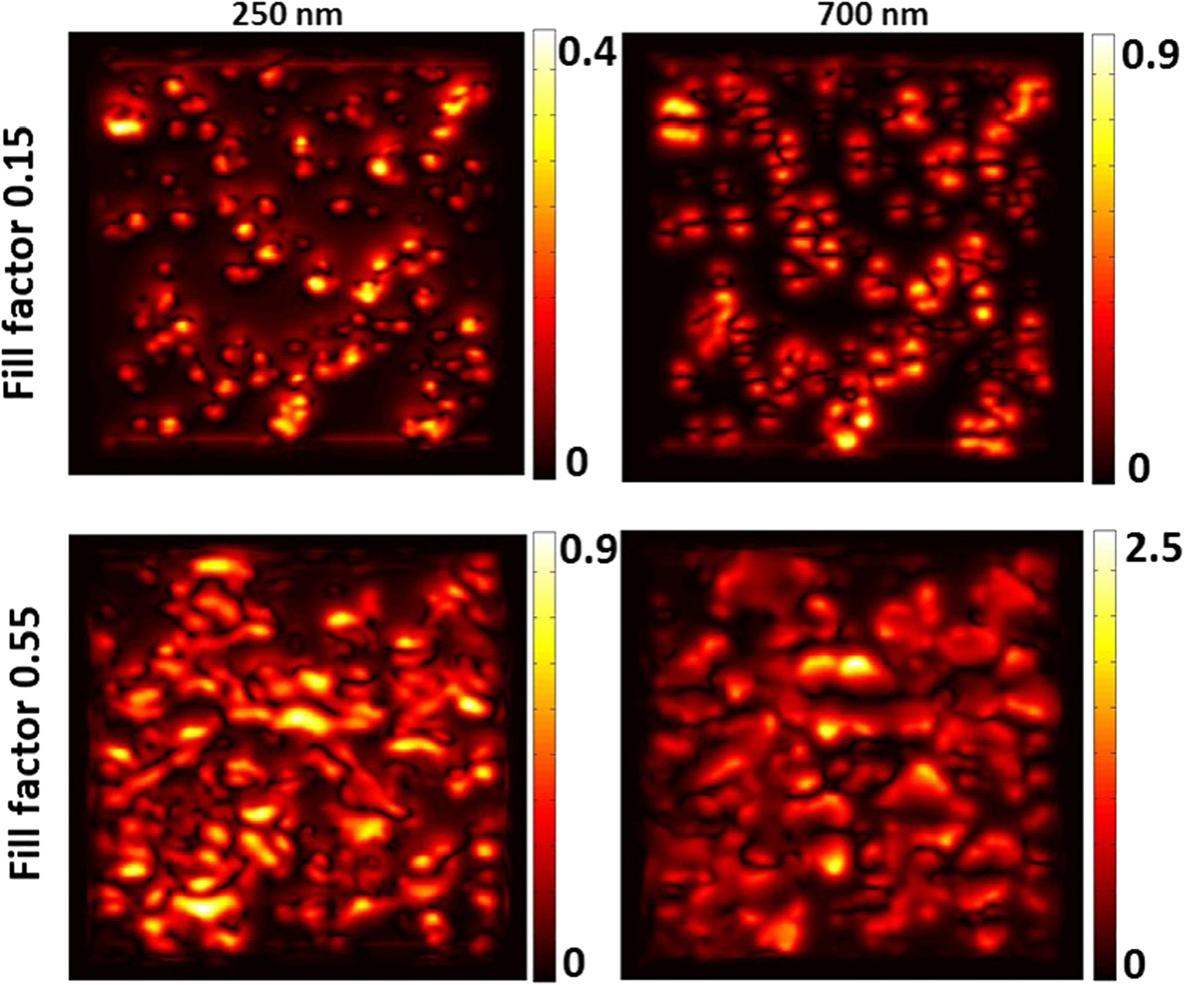
**Calculated local intensity normalized with respect to the incident intensity at different wavelengths.**

The calculated intensity maps (Figure 3) indicate that optical properties of films strongly depend on their local morphology. There are regions of the localization of strongly enhanced electromagnetic fields usually called ‘hot spots’ (brighter areas in Figure 3 correspond to the points where stronger enhancement is observed). This result is consistent with previous calculations performed in the frame of the DDA method using AFM images as initial data [15]. It is worth to be remarked that in the UV (250 nm), one observes a larger number of more intensive ‘hot spots’, while at 700 nm, the intensity is more evenly distributed with a smaller number of strongly enhanced regions.

**Figure 4:**
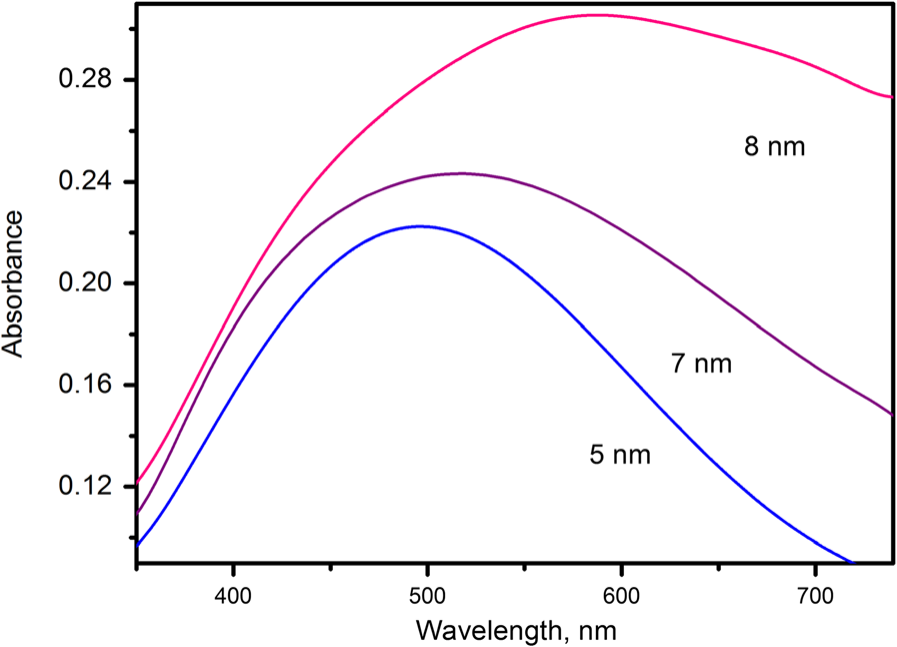
**Normalized absorbance of nanostructured Ag films with different mass thicknesses.**

For silver films on glass, the plasmon resonant absorption is expected in the middle of the visible range [16]. Performed absorption measurements on obtained films are in line with theoretical prediction and previous reports. As depicted in Figure 4, there is a strong absorption band peaked at 496 nm for the film with 5-nm mass thickness. The structure of the film determines the position and shape of the peak. The absorption band broadens as mass thickness increases, and its peak position exhibits red shift (516 nm for 7-nm film and 587 nm for 8-nm film).

Bandwidth is governed by the silver particle size distribution. Silver particles tend to aggregate in clusters and the average size of individual cluster increases with film thickness, leading to broader absorption band.

**Figure 5:**
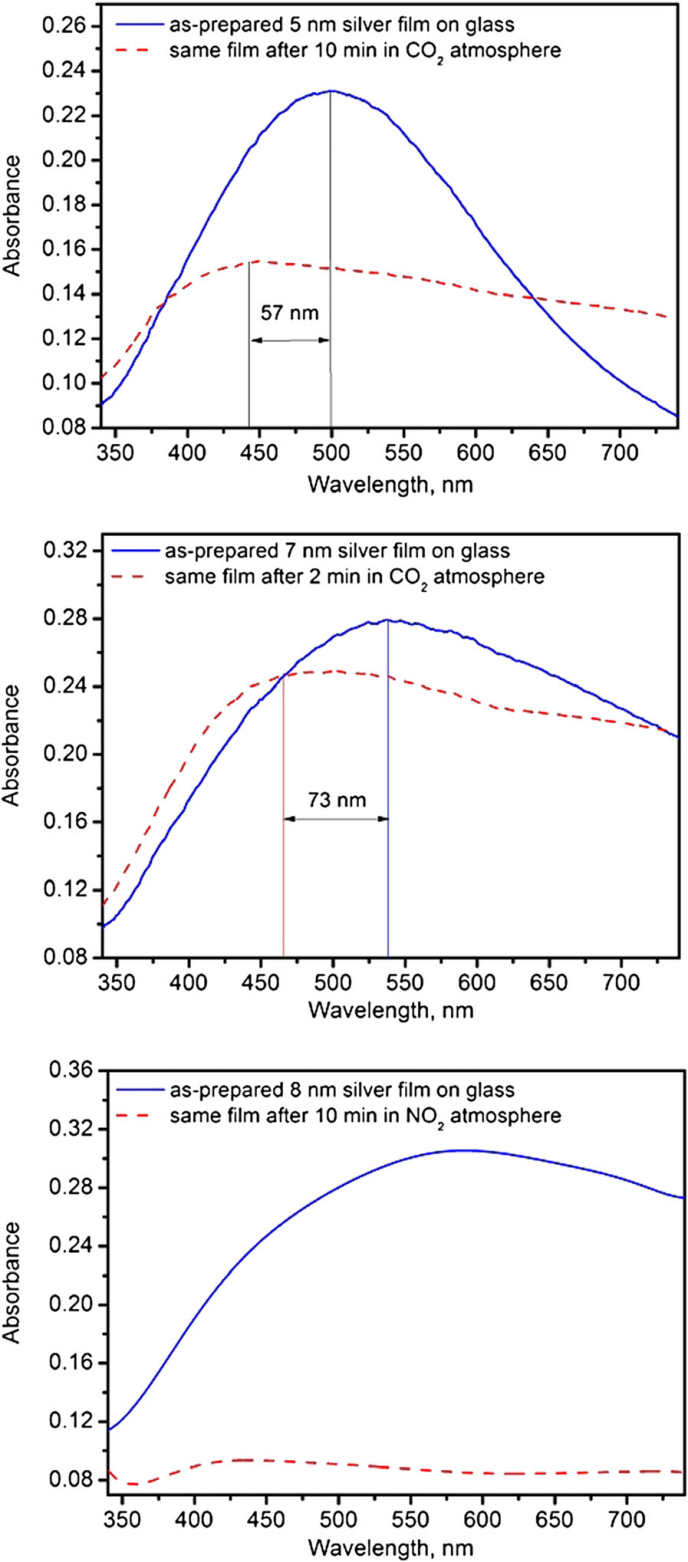
**Influence of nitrogen dioxide (bottom graph) and carbon dioxide environment on the optical absorption of silver films.**

Measurements of the optical absorption of films in different environments confirmed that the presence of gas molecules affects the absorption spectra. The results are presented in Figure 5. It was established that exposing the sample to CO_2_ leads to a slight blue shift of the absorption peak, band broadening, and a decrease in the maximum absorption level. The longer is the exposure time, the lower is the absorption at the peak position. The shift depends on the morphology on the film and is larger for the thicker films (or films with larger silver clusters). The effect of NO_2_ is considerably stronger in the sense that resonant absorption completely disappears.

We assume that silver atoms react with neutral carbon dioxide to form Ag(CO)_*n*_ complexes. Therefore, the amount of silver particles/clusters interacting with the incoming light decreases making the plasmon resonance less pronounced. At the same time, the contribution of created complexes is reflected in broadening of the spectra and overall decrease of absorption level. In case of NO_2_, the oxidizing reaction is possible, eventually leading to the formation of Ag^+^ cations, so the resonance related to neutral Ag^0^ atoms is not observed.

## Conclusions

The morphology of the Ag films was reconstructed using statistical algorithms to resemble the morphology of real random structures, obtained by thermal deposition. The approach is suitable for film growth simulation and for film parameter calculations. Through electrodynamical calculations, it was shown that the local field amplification is well pronounced in nanostructured ultrathin silver films with island structure. From obtained intensity maps, it follows that light enhancement at the nanoscale in investigated structures should be more efficient in the UV and blue spectral region. Optical studies show the sensitivity of the visible absorption of silver nanostructured films to the surrounding atmosphere which can be potentially used to detect noxious gases such as CO_2_ or NO_2_.
